# Indexed oxygen delivery during pediatric cardiopulmonary bypass is a modifiable risk factor for postoperative acute kidney injury

**DOI:** 10.1051/ject/2023029

**Published:** 2023-09-08

**Authors:** Molly Dreher, Jungwon Min, Constantine Mavroudis, Douglas Ryba, Svetlana Ostapenko, Richard Melchior, Tami Rosenthal, Muhammad Nuri, Joshua Blinder

**Affiliations:** 1 Department of Cardiovascular Perfusion, Children’s Hospital of Philadelphia Philadelphia PA 19104 USA; 2 Department of Biomedical and Health Informatics, Children’s Hospital of Philadelphia Philadelphia PA 19104 USA; 3 Children’s Hospital of Philadelphia, Cardiac Center, Division of Cardiothoracic Surgery Philadelphia PA 19104 USA; 4 Information Services Department, Children’s Hospital of Philadelphia Philadelphia PA 19104 USA; 5 Stanford University School of Medicine, Lucile Packard Children’s Hospital, Department of Pediatrics, Division of Pediatric Cardiology Palo Alto CA 94304 USA

**Keywords:** Acute kidney injury, Indexed oxygen delivery, Cardiopulmonary bypass, Pediatric, Cardiac surgery

## Abstract

*Background*: Acute kidney injury after pediatric cardiac surgery is a common complication with few established modifiable risk factors. We sought to characterize whether indexed oxygen delivery during cardiopulmonary bypass was associated with postoperative acute kidney injury in a large pediatric cohort. *Methods*: This was a retrospective analysis of patients under 1 year old undergoing cardiac surgery with cardiopulmonary bypass between January 1, 2013, and January 1, 2020. Receiver operating characteristic curves across values ranging from 260 to 400 mL/min/m^2^ were used to identify the indexed oxygen delivery most significantly associated with acute kidney injury risk. *Results*: We included 980 patients with acute kidney injury occurring in 212 (21.2%). After adjusting for covariates associated with acute kidney injury, an indexed oxygen delivery threshold of 340 mL/min/m^2^ predicted acute kidney injury in STAT 4 and 5 neonates (area under the curve = 0.66, 95% CI = 0.60 – 0.72, sensitivity = 56.1%, specificity = 69.4%). An indexed oxygen delivery threshold of 400 mL/min/m^2^ predicted acute kidney injury in STAT 1-3 infants (area under the curve = 0.65, 95% CI = 0.58 – 0.72, sensitivity = 52.6%, specificity = 74.6%). *Conclusion*: Indexed oxygen delivery during cardiopulmonary bypass is a modifiable variable independently associated with postoperative acute kidney injury in specific pediatric populations. Strategies aimed at maintaining oxygen delivery greater than 340 mL/min/m^2^ in complex neonates and greater than 400 mL/min/m^2^ in infants may reduce the occurrence of postoperative acute kidney injury in the pediatric population.

## Introduction

Acute kidney injury (AKI) occurs in up to 64% of pediatric patients undergoing congenital heart surgery with varying incidence depending on the subject population and AKI definition [1–5]. While historical data suggests an association between AKI and short-term morbidity and mortality, more recent data from the NEPHRON collaborative indicates that only severe cardiac surgery-associated acute kidney injury (CS-AKI) was associated with mortality [6]. Investigators postulate that existing AKI definitions may not accurately describe the breadth of pediatric CS-AKI [6]. Nevertheless, multiple other studies found clinically important CS-AKI associations with poor clinical outcomes [2, 4, 7, 8].

While existing data identified multiple non-modifiable CS-AKI risk factors including longer cardiopulmonary bypass (CPB) duration, use of deep hypothermic circulatory arrest (DHCA), age less than 1 year, single ventricle physiology and high preoperative serum creatinine, few modifiable risk factors have been implicated [1, 2, 4–6, 9, 10]. Data from several adult studies found that indexed oxygen delivery (DO_2_i) during CPB is associated with CS-AKI [11–14]. DO_2_i is the product of CPB flow and arterial blood oxygen content indexed to body surface area (BSA). Ranucci et al. identified 272 mL/min/m^2^ as the nadir DO_2_i necessary to prevent postoperative acute renal failure after coronary artery bypass [11]. Subsequent data in adults identified that targeting DO_2_i values ranging from >225 to 300 mL/min/m^2^ may reduce AKI risk [12–18].

Less DO_2_i data exists in pediatric cardiac surgery [19–21]. Reagor reported that subjects managed with higher CPB flow rates and higher DO_2_i (mean DO_2_i of 335 mL/m/m^2^) had lower CS-AKI incidence [19]. In a recent cohort of 83 infants, a DO_2_i nadir > 353 mL/min/m^2^ was associated with lower CS-AKI risk [20]. In their retrospective analysis of 396 patients under 18 years old, Hayward and colleagues found that cumulative time spent below a DO_2_i of 350 mL/min/m^2^ was associated with AKI risk [21]. These pediatric studies demonstrated considerably higher critical DO_2_i values than the adult data. This finding may be attributed to higher oxygen consumption rates in children compared to adults, especially in young infants and neonates [20–22].

Since there are few established modifiable CS-AKI risk factors, we sought to characterize the role of DO_2_i on postoperative CS-AKI in a large infant (<1 year of age) sample. We hypothesized that longer exposure to lower DO_2_i is associated with CS-AKI and other clinical outcomes. We hope to establish intraoperative DO_2_i targets to limit the impact of clinically significant CS-AKI on this patient population.

## Materials and methods

### Study design and population

All patients under 1-year-old undergoing CPB-assisted cardiac surgery between January 1, 2013, and January 1, 2020, were assessed for eligibility. This time frame was selected due to uniformity in surgical and perfusion staff and techniques. Patients with incomplete Society of Thoracic Surgeons-European Association for Cardio-Thoracic Surgery (STAT) mortality categorization, inadequate serum creatinine, and urine output data to determine AKI status were excluded. Additionally, patients requiring extracorporeal membrane oxygenation (ECMO) preoperatively or within six days of surgery were excluded from the analysis given the increased risk of AKI within this patient population.

The cohort was divided into 4 subgroups based on age and surgical complexity (categorized using STAT mortality categories [23]): neonates (<31 days old) in STAT categories 1–3, neonates in STAT categories 4 and 5, infants (31 days – 1 year) in STAT categories 1–3, and infants in STAT categories 4 and 5. The subgroups were created in an attempt to minimize heterogeneity in our patient population by separating higher-risk patients (i.e. neonates, STAT categories 4 and 5) from lower-risk patients (i.e. infants, STAT categories 1–3). This study merited exemption by Children’s Hospital of Philadelphia’s institutional review board (IRB# 20-017791, effective October 20, 2021).

### Cardiopulmonary bypass technique

Cardiopulmonary bypass was conducted in accordance with the established institutional protocol. All circuits consisted of a Terumo Capiox^®^ FX05 oxygenator (Terumo Corporation, Tokyo, Japan) with a ¼″ arterial roller pump boot. The total prime volume was dependent on the size of the arterial-venous loop, which was selected based on patient weight (Supplemental Table 1). All circuits were primed with Plasma-Lyte A (Baxter Healthcare Corporation, Deerfield, IL), 25% albumin (12.5 g), heparin (500 units), sodium bicarbonate (1 meq/kg), furosemide (1 mg/kg), cefazolin (25 mg/kg), and aminocaproic acid (250 mg/kg) or tranexamic acid (2 mg/kg). If the circuit was blood-primed, additional calcium gluconate (450 mg) and sodium bicarbonate (5 mEq) were added. Methylprednisolone (30 mg/kg) was included in the CPB prime up until November 2013 [24].

Target hematocrit values were age-dependent with a nadir hematocrit of 30% for neonates and 25% for all other patients. Fresh whole blood (<48 h old) or fresh red blood cells (<7 days old) were utilized to maintain the desired hematocrit. Every CPB circuit included a Capiox^®^ HC05 hemoconcentrator (Terumo Corporation, Tokyo, Japan) for volume management via conventional ultrafiltration during bypass. Modified ultrafiltration was utilized following bypass termination on all patients for hemoconcentration and removal of inflammatory mediators.

CPB flow rates were maintained between 125 and 175 mL/kg/min for patients under 31 days of age and between 100 and 150 mL/kg/min for patients 31 days to one year of age. The nasopharyngeal (NP) temperature was typically maintained between 34 °C and 37 °C for cases not requiring deep hypothermic circulatory arrest (DHCA) (18 °C). pH-stat blood gas management was utilized during the cooling phase of all cases requiring hypothermia. Alpha-stat blood gas management was used during the rewarming phase as well as cases performed at normothermia. The arterial partial pressure of oxygen (PaO_2_) was maintained between 150 and 300 mmHg for all cases.

### Data collection and calculations

Demographic information, clinical data, and laboratory data were collected from the hospital medical record (Epic Systems Corporation, Vernona, WI). CardioAccess^®^ (CardioAccess, Inc., Fort Lauderdale, FL) supplied additional demographics, intensive care unit (ICU) and hospital length of stay (LOS) information, Society of Thoracic Surgeons (STS)-defined preoperative risk factors and postoperative complications.

The electronic perfusion record provided cardiac support times, transfusion data, ultrafiltration volumes, and CPB data. Continuous CPB data was automatically captured every 30 s during the bypass run by the JOCAP^®^ XL (MAQUET Cardiopulmonary GmbH, Rastatt, Germany) electronic charting system until April 2018 at which point data were captured by the Epic^®^ medical record (every 60 s). Continuous data included: pump flow, venous and arterial blood gas values, and NP temperature. PaO_2_, arterial oxygen saturation (SaO_2_), hemoglobin, and venous oxygen saturation (SvO_2_) were continuously measured during CPB using the CDI500 Blood Parameter Monitoring System (Terumo Cardiovascular Systems, Ann Arbor, MI). CPB data collected between the start and end of DHCA were excluded from the analysis.

DO_2_i was not actively measured during the time period represented by this study. Instead, pump flow, hemoglobin, SaO_2_ and PaO_2_ data were used to retrospectively calculate DO_2_i using the formula: DO_2_i (mL/min/m^2^) = 10 × pump flow index (L/min/m^2^) × [(hemoglobin (g/dL) × SaO_2_ (%) × 1.36) + (PaO_2_ (mmHg) × 0.003)]. For each patient, DO_2_i was calculated for every 30–60 s of bypass. Area under the curve (AUC) values were then calculated using incremental threshold DO_2_i values ranging from 260 to 400 mL/min/m^2^ (DO_2_i^260^, DO_2_i^280^, DO_2_i^300^,, DO_2_i^320^, DO_2_i^340^, DO_2_i^360^, DO_2_i^380^, DO_2_i^400^). The AUC accounts for time and depth below a threshold DO_2_i value and is more representative of cumulative oxygen debt compared to nadir or median DO_2_i values [16, 18]. We excluded outliers (<3%, by mean + 3*standard deviation) in AUC DO_2_i^260−400^ across the four age-STAT mortality category subgroupings.

### Study outcomes

The primary outcome of this study was postoperative CS-AKI, which was characterized using the Kidney Disease: Improving Global Outcomes (KDIGO) and neonatal KDIGO classification systems [25, 26]. Patients were designated as having CS-AKI if they met the criteria for any stage (1–3) within 7 days of surgery (Supplemental Tables 2 and 3).

Secondary outcomes were: peak cumulative fluid overload, ICU and hospital LOS, in-hospital mortality, and STS postoperative complications including low cardiac output syndrome, cardiac arrest, multisystem organ failure, stroke, and unplanned interventions. We defined peak cumulative fluid overload as the maximum sum of fluid balance during the first three postoperative days. Time to negative fluid balance was defined as the first postoperative day with a negative fluid balance.

### Statistical analysis

Patient demographics and preoperative and operative characteristics were compared by AKI status using *t*-tests, Mann–Whitney *U* tests, chi-square tests, and Fisher’s exact tests. To identify the most significant DO_2_i associated with AKI, we used receiver operating characteristic (ROC) analysis across DO_2_i^260^ – DO_2_i^400^ after adjusting for CPB time (>90 min, ≤90 min), DHCA use (yes/no), nadir NP temperature, intraoperative transfusion (yes/no), patient race (White, Black, other), ethnicity (Hispanic, non-Hispanic) and preterm gestational age (yes/no, neonates only).

After identifying the DO_2_i with the highest AUC for AKI, we used Youden’s index (best sensitivity value + [specificity − 1]) to determine the best AUC cut-off value in DO_2_i to predict AKI risk. Finally, we examined associations between the best cut-off in DO_2_i with secondary outcomes using regression and logistic regression models after adjusting for the set of covariates above. The model with all covariates had the best predictive power by testing different sets of covariates in the multivariate models [27, 28]. All statistical tests are two-sided, and a *p-*value of 0.05 determined statistical significance. All analyses were conducted using SAS version 9.4 (SAS Institute, Cary, NC).

## Results

### Patient characteristics

**Figure 1:**
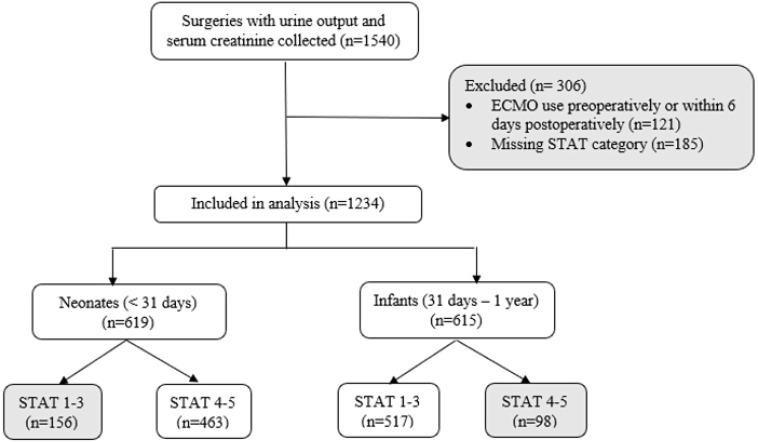
Study flow diagram.

We identified 1540 patients under one year of age with the necessary urine output and serum creatinine data to determine AKI status. Patients requiring ECMO preoperatively or within six days of surgery (*n* = 121) and those missing STAT mortality categorization (*n* = 185) were excluded from the analysis (Figure 1).

Neonates in STAT categories 1–3 (*n* = 156) and infants in STAT categories 4–5 (*n* = 98) did not show a significantly larger DO_2_i AUC in the AKI group than in the non-AKI group in *t*-tests and adjusted regression analyses. Demographic data for the remaining two subgroups: neonates in STAT categories 4 and 5 (*n* = 463) and infants in STAT categories 1–3 (*n* = 517) are presented in Table 1 (Supplemental Table 4 presents all four subgroups). Single ventricle physiology was present in 37.1% of the patient population. Any CS-AKI occurred after 212 procedures (21.2%) and severe CS-AKI (stage 3) occurred after only 16 procedures (1.86%).

**Table 1:** Demographics and preoperative characteristics by AKI for STAT 4 & 5 neonates (*n* = 463) and STAT 1–3 infants (*n* = 517).

	All (*n* = 980)	Non-AKI (*n* = 768)	AKI (*n* = 212)	*p* value
Frequency (%)				
Age at surgery				
<31 days	463 (47.2)	336 (43.8)	127 (59.9)	<0.001
31 days – ≤1 year	517 (52.8)	432 (56.3)	85 (40.1)	
Male	550 (56.1)	421 (54.8)	129 (60.9)	0.11
Race				0.07
White	536 (54.7)	420 (54.7)	116 (54.7)	
Black	134 (13.7)	114 (14.8)	20 (9.4)	
Other	310 (31.6)	234 (30.5)	76 (35.9)	
Hispanic or Latino	165 (16.8)	122 (15.9)	43 (20.3)	0.12
Gestational age < 37 weeks	132 (13.7)	99 (13.1)	33 (15.9)	0.29
Ventricular physiology				0.24
Single ventricle	364 (37.1)	278 (36.2)	86 (40.6)	
Two ventricles	616 (62.9)	490 (63.8)	126 (59.4)	
Max STAT score				<0.001
1	191 (19.5)	172 (22.4)	19 (9)	
2	211 (21.5)	171 (22.3)	40 (18.9)	
3	115 (11.7)	89 (11.6)	26 (12.3)	
4	296 (30.2)	227 (29.6)	69 (32.6)	
5	167 (17)	109 (14.2)	58 (27.4)	
STS risk factors				
Chromosomal anomaly	328 (33.5)	260 (33.9)	68 (32.1)	0.62
Preoperative mechanical ventilation	130 (13.3)	82 (10.7)	48 (22.6)	<0.001
Emergency	2 (0.2)	1 (0.1)	1 (0.5)	0.38
Cardiopulmonary Resuscitation	7 (0.7)	7 (0.9)	2 (0.9)	0.35
Colostomy	8 (0.8)	6 (0.8)	1 (0.5)	0.68
Endocarditis	2 (0.2)	1 (0.1)	10 (4.7)	0.38
Gastrostomy	34 (3.5)	24 (3.1)	2 (0.9)	0.26
Necrotizing enterocolitis treated medically	11 (1.1)	9 (1.2)	2 (0.9)	1.00
Atrioventricular block	6 (0.6)	4 (0.5)	2 (0.9)	0.61
Mechanical circulatory support	4 (0.4)	2 (0.3)	1 (0.5)	0.20
Renal dysfunction	3 (0.3)	2 (0.3)	5 (2.4)	0.51
Seizure	27 (2.8)	22 (2.9)	3 (1.4)	0.69
Sepsis	8 (0.8)	5 (0.7)	1 (0.5)	0.38
Shocking occurring at reoperation	3 (0.3)	2 (0.3)	9 (4.3)	0.51
Shock resolved at time of operation	21 (2.1)	12 (1.6)	16 (7.6)	0.02
Stroke	51 (5.2)	35 (4.6)	68 (32.1)	0.08
Mean (SD)				
Age at surgery in years	0.0 (0.5)	0.0 (0.1)	0.1 (1.0)	0.34
Weight at surgery in kg	4.4 (2.0)	4.4 (1.5)	4.2 (3.3)	0.58
BSA at surgery in m^2^	0.3 (0.1)	0.3 (0.1)	0.3 (0.1)	0.12
Gestational age	38.0 (2.2)	38.0 (2.1)	37.7 (2.3)	0.07
Preoperative creatinine in mg/dL	0.4 (0.4)	0.4 (0.5)	0.4 (0.2)	0.93
Total number of 16 STS preoperative risk factors	0.7 (0.9)	0.6 (0.8)	0.8 (1.0)	0.01

Variables were compared using *t*-test, *χ*^2^ test, and Fisher’s exact test. Data is presented as mean (standard deviation) or as number (percent). BSA: body surface area; SD: standard deviation; STAT: Society of Thoracic Surgeons-European Association for Cardio-Thoracic Surgery; STS: Society of Thoracic Surgeons.

### Preoperative CS-AKI risk factors

Preoperative CS-AKI risk factors are summarized in Table 1. There was no difference in the incidence of CS-AKI in patients with single ventricle physiology compared to patients with two ventricles (23.6% vs. 20.5%, *p* = 0.24). Of the STS-defined preoperative risk factors, the need for mechanical ventilation (*p* < 0.001) and preoperative shock (*p* = 0.02) were associated with CS-AKI. There was no difference in preoperative creatinine levels between patients with or without CS-AKI.

### Intraoperative CS-AKI risk factors

Intraoperative characteristics are presented in Table 2 (Supplemental Table 5 presents all four subgroups). CS-AKI patients had longer cardiac support times: CPB time (84 min vs. 67 min, *p* < 0.001) and cross-clamp time (50 min vs. 34 min, *p* < 0.001). For CS-AKI patients, the mean CPB cardiac index was lower compared to the non-AKI group (2.48 L/min/m^2^ vs. 2.53 L/min/m^2^, *p* = 0.03). AKI patients had lower nadir nasopharyngeal (NP) temperature (33.0 °C vs. 34.0 °C, *p* < 0.001), lower median oxygen consumption (VO_2_) (57.7 mLO_2_/min/m^2^ vs. 77.3 mLO_2_/min/m^2^, *p* < 0.001), higher median venous oxygen saturation (81% vs. 77%, *p* < 0.001), and lower oxygen extraction ratio (OER) (16.8% vs. 21.5%, *p* < 0.001) compared to the non-AKI group. These indices of decreased oxygen demand may be attributed to the higher use of DHCA in patients with CS-AKI (52.4% vs. 40.0%, *p* < 0.001). DHCA patients had lower VO_2_ (41.4 mLO_2_/min/m^2^ vs. 96.2 mLO_2_/min/m^2^), higher SvO_2_ (86.5% vs. 71.7%) and lower OER (12.0% vs. 26.3%) (*p* < 0.001 for all comparisons).

**Table 2:** Operative characteristics by AKI for STAT 4 & 5 neonates (*n* = 463) and STAT 1–3 infants (*n* = 517).

	All (*n* = 980)	Non-AKI (*n* = 768)	AKI (*n* = 212)	*p* value
CPB time (min)	70.0 (41.0)	67.0 (42.0)	84.0 (45.5)	<0.001
Cross clamp time (min)	37.0 (33.0)	34.0 (32.0)	50.0 (39.0)	<0.001
Use of DHCA	562 (42.7)	307 (40.0)	111 (52.4)	<0.001
Nadir NP temperature (°C)	34.0 (7.1)	34.0 (7.0)	33.0 (15.0)	<0.001
Nadir hematocrit	25.0 (4.0)	25.0 (4.0)	25.0 (4.0)	0.03
CPB cardiac index (L/min/m^2^)	2.5 (0.4)	2.5 (0.4)	2.5 (0.5)	0.03
Median VO_2_ (mL/min/m^2^)	72.3 (60.8)	77.3 (61.3)	57.7 (58.5)	<0.001
Median SvO_2_ (%)	78.0 (16.5)	77 (16)	81 (16)	<0.001
Median PO_2_ (mmHg)	289.0 (49.5)	289 (48.9)	290 (54.5)	0.34
Median oxygen extraction ratio	20.5 (16.1)	21.5 (15.6)	16.8 (15.5)	<0.001
Intraoperative transfusion (y/n)	754 (76.9)	572 (74.5)	182 (85.9)	<0.001
Whole blood (mL/kg)	0 (8.8)	6.9 (18.8)	11.6 (26)	0.001
RBC (mL/kg)	0 (0)	0 (4.9)	0 (23.5)	<0.001
FFP (mL/kg)	0 (0)	0 (0)	0 (0)	<0.001
Platelets (mL/kg)	0 (0)	0 (0)	0 (8.3)	0.003
Use of modified ultrafiltration	949 (96.8)	744 (96.9)	205 (96.7)	0.97
Total UF volume (mL/kg)	90.9 (47.6)	88.5 (45.4)	99.4 (56.2)	<0.001
Total UF volume ≥ 119.9 mL/kg	223 (22.8)	150 (19.6)	73 (34.4)	0.001
AUC < DO_2_i^400^	1978.5 (2651.5)	1882.8 (2483)	2533.7 (3858.6)	<0.001
AUC < DO_2_i^380^	1363.3 (2085.5)	1308.1 (1941.7)	1854.6 (3091.1)	<0.001
AUC < DO_2_i^360^	933.1 (1541.4)	884.7 (1390)	1210.8 (2266.5)	<0.001
AUC < DO_2_i^340^	634.7 (1008.3)	585.4 (923.9)	796.1 (1521.6)	<0.001
AUC < DO_2_i^320^	411.2 (711.6)	378.1 (645.4)	564.4 (981.3)	<0.001
AUC < DO_2_i^300^	283.5 (497.9)	274.5 (472)	353.3 (633.5)	<0.001
AUC < DO_2_i^280^	223.7 (398.9)	213.9 (357.6)	258.4 (452.3)	0.003
AUC < DO_2_i^260^	167.6 (320.7)	158.3 (299)	198.2 (424.2)	0.01

Variables were compared using the Mann–Whitney *U* test or *χ*^2^ test. Data are presented as median (IQR) because of non-normal distribution or as number (percent). AUC: area under the curve; CPB: cardiopulmonary bypass; DHCA: deep hypothermic circulatory arrest; DO_2_i: indexed oxygen delivery; FFP: fresh frozen plasma; NP: nasopharyngeal; PO_2_: partial pressure of oxygen; RBC: red blood cells; SvO_2_: venous oxygen saturation; UF: ultrafiltration; VO_2_: oxygen consumption.

The nadir hematocrit was significantly lower in CS-AKI patients (23.84 ± 4.65% vs. 24.38 ± 5.10%, *p* = 0.03). Nearly 86% of CS-AKI patients received intraoperative blood products versus 74.5% of patients without CS-AKI (*p* < 0.001) (Table 2). While we identified no difference in the use of modified ultrafiltration between the groups, patients with CS-AKI had greater total ultrafiltration volume removed (99.4 mL/kg vs. 88.5 mL/kg, *p* < 0.001). Additionally, significantly more patients with CS-AKI had > 119.9 mL/kg of total ultrafiltration removed during bypass (34.4% vs. 19.6%, *p* = 0.001).

**Figure 2:**
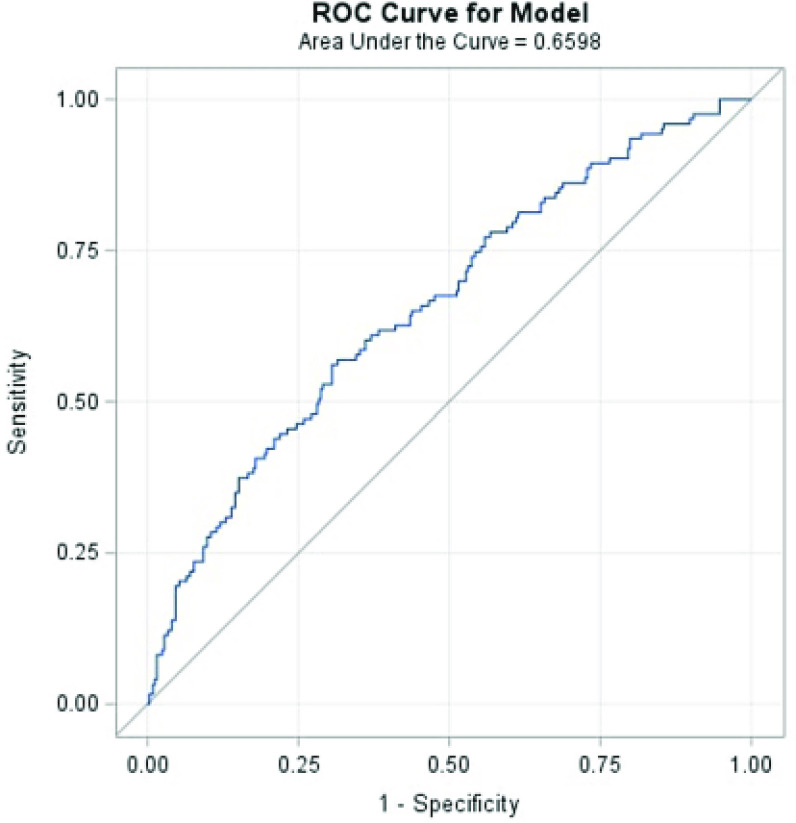
Adjusted receiver operating characteristic analysis of area under the curve (AUC) for DO_2_i^340^ and any stage CS-AKI for neonates in STAT categories 4 and 5, *n* = 463 patients. A logistic regression model after adjusting for CPB time (> 90 min, ≤ 90 min), DHCA use (yes/no), nadir NP temperature, intraoperative transfusion (yes/no), patient race (White, Black, other), ethnicity (Hispanic, non-Hispanic), and preterm gestational age (yes/no), DO_2_i^340^ had the highest AUC of CS-AKI, 0.66 (95% CI = 0.60 – 0.72, sensitivity = 56.1%, specificity = 69.4%).

**Figure 3:**
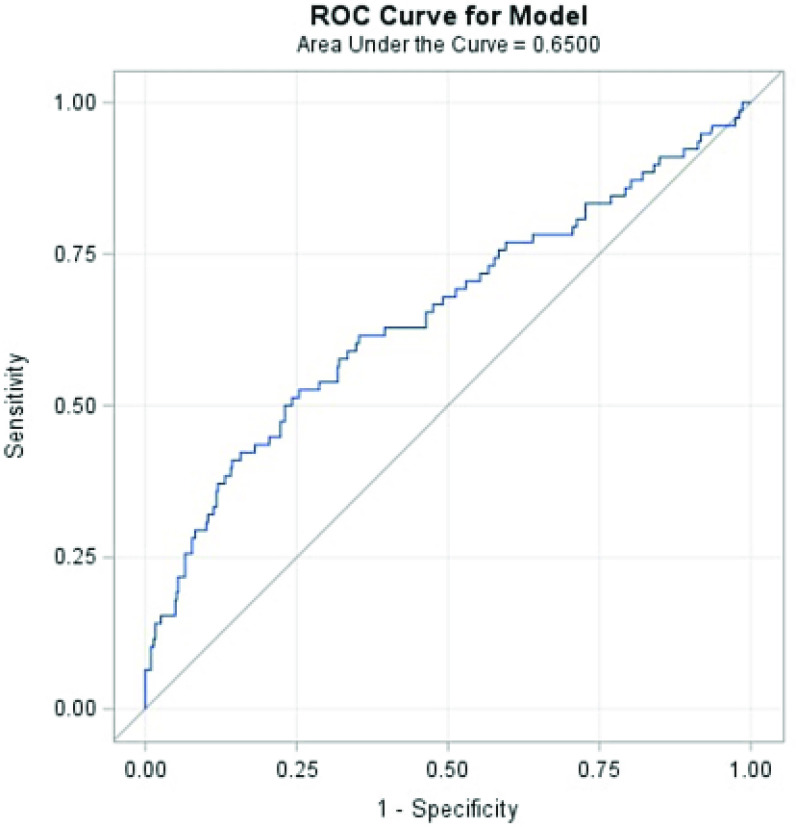
Adjusted receiver operating characteristic analysis of area under the curve (AUC) for DO_2_i^400^ and any stage CS-AKI for infants in STAT categories 1–3, *n* = 517 patients. A logistic regression model after adjusting for CPB time (>90 min, ≤90 min), DHCA use (yes/no), nadir NP temperature, intraoperative transfusion (yes/no), patient race (White, Black, other), and ethnicity (Hispanic, non-Hispanic), DO_2_i^400^ had the highest AUC of CS-AKI, 0.65 (95% CI = 0.58 – 0.72, sensitivity = 52.6%, specificity = 74.6%).

AUC values for DO_2_i^260−400^ greater than mean + 3SD (outliers) were treated as missing in the analysis. STAT 4 and 5 neonates (*n* = 463) had 450–451 observations per DO_2_i threshold and STAT 1–3 infants (*n* = 517) had 506–509 observations per DO_2_i threshold. The median AUC value calculated for each oxygen delivery threshold (DO_2_i^260^ – DO_2_i^400^) was significantly higher in patients with any stage of CS-AKI (Table 2). After adjusting for covariates in the neonatal STAT 4 and 5 subgroup, ROC analysis demonstrated an AUC < DO_2_i^340^ was a fair predictor of AKI (AUC = 0.66, 95% CI = 0.60 – 0.72, sensitivity = 56.1%, specificity = 69.4%) with a cutoff value of 660.59 (Figure 2). For the infant STAT 1–3 subgroup, ROC analysis demonstrated an AUC < DO_2_i^400^ was a fair CS-AKI predictor (AUC = 0.65, 95% CI = 0.58 – 0.72, sensitivity = 52.6%, specificity = 74.6%) with a cutoff value of 3709.21 (Figure 3). Neither AUC < DO_2_i^340^ in neonates nor AUC < DO_2_i^400^ in infants was associated with ICU and hospital length of stay, fluid overload, and time to negative fluid balance.

## Discussion

Our results demonstrate that DO_2_i on CPB may represent a modifiable risk factor for postoperative CS-AKI in the pediatric population. We identified 340 mL/min/m^2^ as the threshold DO_2_i associated with reduced CS-AKI risk in neonates undergoing STAT category 4 and 5 procedures and 400 mL/min/m^2^ as the threshold DO_2_i associated with reduced CS-AKI risk in infants undergoing STAT category 1–3 procedures. We did not identify a relationship between DO_2_i and CS-AKI in STAT 1–3 neonates and STAT 4 and 5 infants, and it is unclear if this is due to lack of effect or to the relatively small sample size of these two subgroups. We also determined cutoff values for the AUC < DO_2_i^340^ (660.59) and AUC < DO_2_i^400^ (3709.21), which are findings not previously described in the pediatric literature. These AUC cutoff values represent a measurable number that can be monitored in real-time with the use of modern perfusion equipment. Our data represents the largest retrospective study exploring the relationship between CPB-indexed oxygen delivery and pediatric postoperative CS-AKI.

CS-AKI remains a common postoperative complication after CPB-assisted pediatric cardiac surgery, occurring in up to 64% of children [1]. While recent neonatal data has shown that only severe AKI (KDIGO stage 3) is associated with increased mortality, historical data found associations between CS-AKI and prolonged mechanical ventilation, increased ICU and hospital LOS, and increased mortality [1–3, 5–7]. Given this clinical significance, identifying ways to minimize CS-AKI may lead to improved outcomes. Our results are consistent with previously reported non-modifiable CS-AKI risk factors including prolonged cardiac support times, use of DHCA, more complex surgery, and younger age [1, 2, 4–6, 8, 9].

Our DO_2_i^340^ and DO_2_i^400^ values are considerably higher than the threshold DO_2_i values reported in early adult studies (225–272 mL/min/m^2^), though more recent adult data found that maintaining DO_2_i ≥ 300 mL/min/m^2^ is associated with reduced CS-AKI risk [11–14, 16, 18]. Our data differs in an important way from the earlier literature. Initial studies collected CPB data in 20- or 30-min intervals and evaluated nadir DO_2_i values. Since oxygen delivery is dynamic and changes throughout the surgical procedure, nadir DO_2_i values may inaccurately reflect oxygen delivery during the totality of the CPB run. More recent studies collected CPB data continuously (every 20 s) which accounted for more dynamic DO_2_i changes. AUC measurements better represent CPB oxygen debt by integrating the time and depth below a threshold DO_2_i value [18].

Our data found a DO_2_i^340^ AUC ≥ 660.59 among complex neonates with CS-AKI and a DO_2_i^400^ AUC ≥ 3709.21 in less complex infants with CS-AKI. Both results indicate that prolonged and lower oxygen delivery is associated with postoperative CS-AKI. The higher threshold DO_2_i value in less complex patients between 31 days and 1 year of age is a surprising finding, not previously reported in literature. This increased DO_2_i in infants may be related to the rise in oxygen consumption that occurs over the first few months of life [22]. However, the lower AUC cutoff value for our neonatal subgroup compared to our infant subgroup (660.59 vs. 3709.21) suggests that complex neonates may have a lower tolerance to decreased oxygen delivery during CPB.

Studies on oxygen delivery and postoperative CS-AKI in the pediatric population are limited. A recent cohort of 396 pediatric patients (<18 years old) undergoing cardiac surgery with CPB found that cumulative time spent under a DO_2_i of 350 mL/min/m^2^ was independently associated with AKI [21]. While this threshold value was similar to our findings, the authors did not report on AUC values, instead focusing on cumulative time rather than both time and depth below the 350 mL/min/m^2^ threshold. In a study examining a small cohort of 83 young children (1 month – 3 years old), the nadir DO_2_i associated with lower CS-AKI risk was 353 mL/min/m^2^ during CPB with mild hypothermia [20]. Interestingly, Gao recently reported significantly lower DO_2_i thresholds during the mildly hypothermic (258 mL/min/m^2^) and rewarming phases (281 mL/min/m^2^) of CPB in their infant population (*n* = 371) [29]. The authors attribute their lower DO_2_i values to a conservative transfusion policy as well as reduced flows secondary to circuit miniaturization but provide no data on either variable. Since both of these recent studies lacked continuous DO_2_i measurements, AUC values were not reported and we cannot infer whether these nadir DO_2_i values reflect the same level of oxygen debt as our AUC values. Identifying an AUC cutoff is critical to modify CS-AKI risk as modern perfusion equipment can display real-time AUC values programmed to a pre-specified DO_2_i threshold, allowing clinicians to monitor and adjust oxygen delivery variables during CPB.

The causative relationship between low DO_2_i and CS-AKI may represent an oxygen supply-demand mismatch. Not only does CPB-induced hemodilution decrease oxygen-carrying capacity, but the neuroendocrine response to CPB leads to renal vasoconstriction and decreased renal blood flow [30]. One principle CPB goal is to balance hemodilution and pump flow to maintain a DO_2_i high enough to prevent anaerobic metabolism and oxidative injury. A recent study by Bojan identified 340 mL/min/m^2^ as the minimum DO_2_i necessary to maintain aerobic metabolism in neonates on CPB [31]. Interestingly, this is the same DO_2_i value associated with decreased AKI risk in our neonatal subgroup.

Given the relatively small contribution of dissolved oxygen, DO_2_i is primarily a factor in pump flow and patient hematocrit. Tadphale investigated postoperative AKI in 151 infants undergoing cardiac surgery and found that a high flow, high hematocrit CPB strategy (CPB flow: 175–200 mL/kg/min, Hct > 32%) reduced AKI rates compared to a lower flow, lower hematocrit strategy (CPB flow: 150 mL/kg/min, Hct > 25%) [32]. While it is understood that higher flow and higher hematocrit increase oxygen delivery, Tadphale et al. did not report DO_2_i values associated with their differing CPB strategies. In an evaluation of two perfusion strategies, Reagor et al. found that the higher DO_2_i (377 ± 42.9 mL/min/m^2^) associated with a higher flow CPB strategy (CI of 3.0 L/min/m^2^ vs. 2.4 L/min/m^2^) reduced the occurrence of all stages of postoperative AKI in a pediatric cohort [19]. Interestingly, the higher flow CPB strategy in this study had a lower nadir hematocrit (25%) compared to the lower flow group (28%), which may indicate the greater importance of increased CPB flow in reducing AKI [19]. Our data also indicated the importance of CPB flow in reducing AKI, as our non-AKI group had a significantly higher CI on CPB (2.53 L/min/m^2^ vs. 2.48 L/min/m^2^, *p* = 0.03).

Data from our study also supports previously published literature that showed higher indexed ultrafiltration volume was associated with increased CS-AKI risk [33]. While this outcome is not directly related to DO_2_i, it does present an additional potentially modifiable CS-AKI risk factor. Our data, however, does not shed new light on fluid overload risk factors as we identified no relationship between DO_2_i and peak cumulative fluid overload or time to negative fluid balance in either patient subgroup.

Our study has several important limitations. This was a single-center, retrospective, observational study, which may limit generalizability and may be confounded by unmeasured center-specific variables. In addition, unmeasured variability in perioperative management and treatment (e.g. nephrotoxic drug use) may have occurred over the 7-year time frame of this study. CPB flow was calculated based on pump speed rather than measured with a flow probe distal to all shunts including the hemoconcentrator and the sampling manifold. Our institutional practice is to increase arterial pump flow to account for blood flow being shunted to the hemoconcentrator pump, however, the sampling manifold shunts continuously, accounting for approximately 0.02 L/min of flow in our neonatal and infant circuit. As a result, our calculated flow values may be slightly higher than the actual flow to the patient. In addition, we did not factor in native patient cardiac output during partial CPB, such as when the cross-clamp was not applied, during CPB initiation and weaning. Given our inability to precisely measure native cardiac output while on bypass, DO_2_i values may be artificially elevated during these brief time periods. We analyzed DO_2_i thresholds in 20 mL/min/m^2^ intervals over a range we felt was sufficient based on previously published literature [11, 16–21]. It is possible that smaller intervals might have provided an even more precise DO_2_i target. Lastly, the KDIGO classification system was chosen due to its frequent use in studies evaluating AKI after cardiac surgery and because it is the only system to have modified criteria for neonates [5, 10, 16, 18, 19, 34]. However, research has identified potential limitations to applying KDIGO criteria to neonates given that not all elevations in serum creatinine or instances of oliguria are of equal clinical importance, which may inadequately predict morbidity and mortality [6].

## Conclusions

Acute kidney injury after pediatric cardiac surgery is a common complication that may negatively impact patient outcomes. While many risk factors for CS-AKI in the pediatric population are non-modifiable, lower indexed oxygen delivery during bypass is a modifiable variable independently associated with postoperative CS-AKI. An AUC < DO_2_i^340^ below 660.59 may reduce the occurrence of postoperative CS-AKI in complex neonates while an AUC < DO_2_i^400^ below 3709.21 may prevent CS-AKI in STAT 1-3 infants. CPB strategies aimed at maximizing intraoperative oxygen delivery may lead to improved patient outcomes. Further prospective research comparing different DO_2_i strategies is warranted to limit intraoperative end-organ dysfunction in children undergoing cardiac surgery.

## Data Availability

The research data associated with this article are included in the article.

## Supplementary material

Supplementary material is available at https://www.ject.edpsciences.org/10.1051/ject/2023029/olm. *Supplemental Table 1*. CPB components and total prime volume. *Supplemental Table 2*. KDIGO staging criteria for acute kidney injury [23]. *Supplemental Table 3*. Neonatal KDIGO staging criteria for acute kidney injury [24]. *Supplemental Table 4*. Demographics and preoperative characteristics by AKI for patients in all STAT categories (*n* = 1234). *Supplemental Table 5*. Operative characteristics by AKI for patients in all STAT categories (*n* = 1234).
