# Supplementary material for: Indexed oxygen delivery during pediatric cardiopulmonary bypass is a modifiable risk factor for postoperative acute kidney injury
Source: J Extra Corpor Technol. 2023 Sep 8;55(3):112–20. doi: 10.1051/ject/2023029 (PMC10487348; doi:10.1051/ject/2023029)
Supplement: Supplementary file 1 — Supplemental Table 1. CPB components and total prime volume. Supplemental Table 2. KDIGO staging criteria for acute kidney injury [23]. Supplemental Table 3. Neonatal KDIGO staging criteria for acute kidney injury [24]. Supplemental Table 4. Demographics and preoperative characteristics by AKI for patients in all STAT categories (n = 1234). Supplemental Table 5. Operative characteristics by AKI for patients in all STAT categories (n = 1234). [file ject-55-112-s1.pdf]

Supplemental Table 1. CPB components and total prime volume.

Patient Weight	Oxygenator	Arterial-Venous Loop	Prime Volume
0 – 4 kg	Terumo FX05	1/8” x 3/16”	165 mL
4.1 – 6 kg	Terumo FX05	3/16” x 3/16”	185 mL
6.1 – 10 kg	Terumo FX05	3/16” x 1/4"	205 mL
10.1 – 13 kg	Terumo FX05	1/4" x 1/4"	225 mL

Supplemental Table 2. KDIGO staging criteria for acute kidney injury [23].

Stage	Serum Creatinine	Urine Output
1	1.5 – 1.9 times baseline or ≥0.3 mg/dL increase within 48 h	<0.5 mL/kg/h for 6-12 h
2	2.0 – 2.9 times baseline	<0.5 mL/kg/h for ≥12 h
3	3.0 times baseline or ≥4.0 mg/dL or initiation of renal replacement therapy	<0.3 mL/kg/h for ≥24 h or anuria for ≥12 h

Supplemental Table 3. Neonatal KDIGO staging criteria for acute kidney injury [24].

Stage	Serum Creatinine	Urine Output
1	1.5 – 1.9 times reference* or ≥0.3 mg/dL increase within 48 h	<0.5 mL/kg/h for 6-12 h
2	2.0 – 2.9 times reference	<0.5 mL/kg/h for ≥12 h
3	3.0 times reference or ≥2.5 mg/dL or initiation of renal replacement therapy	<0.3 mL/kg/h for ≥24 h or anuria for ≥12 h

*Reference serum creatinine defined as lowest previous value

Supplemental Table 4. Demographics and preoperative characteristics by AKI for patients in all STAT categories (n=1234).

	**All (n=1234)**	**Non-AKI (n=971)**	**AKI (n=263)**	***p* value**
**Frequency (%)**				
Age at surgery				
< 31 days	619 (50.2)	465 (47.9)	154 (58.6)	0.002
31 days- ≤ 1 year	615 (49.8)	506 (52.1)	109 (41.4)	
Male	707 (57.3)	550 (56.6)	157 (59.7)	0.37
Race				0.05
White	671 (54.4)	531 (54.7)	140 (53.2)	
Black	157 (12.7)	133 (13.7)	24 (9.1)	
Other	406 (32.9)	307 (31.6)	99 (37.6)	
Hispanic or Latino	218 (17.7)	161 (16.6)	57 (21.7)	0.05
Gestational age <37 weeks	186 (15.1)	140 (14.4)	46 (17.4)	0.21
Ventricular Physiology				0.23
Single ventricle	399 (32.3)	306 (31.5)	93 (35.4)	
Two ventricles	835 (67.7)	665 (68.5)	170 (64.6)	
Max STAT score				<0.001
1	197 (16)	177 (18.2)	20 (7.6)	
2	241 (19.5)	195 (20.1)	46 (17.5)	
3	235 (19)	189 (19.5)	46 (17.5)	
4	389 (31.5)	297 (30.6)	92 (35)	
5	172 (13.9)	113 (11.6)	59 (22.4)	
STS risk factors				
Chromosomal anomaly	387 (31.4)	302 (31.1)	85 (32.3)	0.70
Preoperative mechanical ventilation	242 (19.6)	172 (17.7)	70 (26.6)	<0.001
Emergency	10 (0.8)	8 (0.8)	2 (0.8)	1.00
Cardiopulmonary Resuscitation	10 (0.8)	10 (1)	2 (0.8)	0.13
Colostomy	11 (0.9)	9 (0.9)	1 (0.4)	1.00
Endocarditis	3 (0.2)	2 (0.2)	10 (3.8)	0.51
Gastrostomy	39 (3.2)	29 (3)	2 (0.8)	0.50
Necrotizing enterocolitis treated medically	14 (1.1)	12 (1.2)	2 (0.8)	0.74
Atrioventricular block	6 (0.5)	4 (0.4)	4 (1.5)	0.61
Mechanical circulatory support	13 (1.1)	9 (0.9)	1 (0.4)	0.49
Renal dysfunction	4 (0.3)	3 (0.3)	7 (2.7)	1.00
Seizure	33 (2.7)	26 (2.7)	3 (1.1)	0.98
Sepsis	9 (0.7)	6 (0.6)	1 (0.4)	0.41
Shocking occurring at reoperation	4 (0.3)	3 (0.3)	11 (4.2)	1.00

Shock resolved at time of operation	43 (3.5)	32 (3.3)	19 (7.2)	0.45
Stroke	66 (5.4)	47 (4.8)	85 (32.3)	0.12
**Mean (SD)**				
Age at surgery in years	0.0 (0.4)	0.0 (0.1)	0.1 (0.9)	0.34
Weight at surgery in kg	4.3 (2.0)	4.3 (1.5)	4.2 (3.0)	0.55
BSA at surgery in m^2	0.3 (0.1)	0.3 (0.1)	0.3 (0.1)	0.42
Gestational age	37.9 (2.2)	37.9 (2.2)	37.6 (2.3)	0.03
Preoperative creatinine in mg/dL	0.4 (0.4)	0.4 (0.4)	0.4 (0.2)	0.44
Total number of 16 STS preoperative risk factors	0.7 (0.9)	0.7 (0.9)	0.8 (1.0)	0.03

Variables were compared using *t*-test, χ^2 test, and Fisher's exact test. Data are presented as mean (standard deviation) or as number (percent). BSA: body surface area; SD: standard deviation; STAT: Society of Thoracic Surgeons-European Association for Cardio-Thoracic Surgery; STS: Society of Thoracic Surgeons.

Supplemental Table 5. Operative characteristics by AKI for patients in all STAT categories (n=1234).

	All (n=1234)	Non-AKI (n=971)	AKI (n=263)	*p* value
CPB time (min)	71 (40)	68 (40)	84 (46)	<0.001
Cross clamp time (min)	41 (35)	40 (35)	50 (40)	<0.001
Use of DHCA	482 (39.1)	361 (37.2)	121 (46.0)	<0.001
Nadir NP temperature (°C)	34 (7)	34.7 (7)	33 (12.6)	<0.001
Nadir hematocrit	25 (4)	25 (4)	25 (4)	0.17
CPB cardiac index (L/min/m^2)	2.5 (0.4)	2.5 (0.4)	2.5 (0.5)	0.01
Median PO_2 (mmHg)	289 (47.5)	289 (48)	286 (51)	0.90
Intraoperative transfusion (y/n)	956 (77.5)	733 (75.5)	223 (84.8)	<0.001
Whole blood (mL/kg)	7.9 (21.7)	7.2 (19.6)	13.9 (27.8)	<0.001
RBC (mL/kg)	0 (10.1)	0 (6.8)	0 (23.7)	<0.001
FFP (mL/kg)	0 (0)	0 (0)	0 (0)	<0.001
Platelets (mL/kg)	0 (0)	0 (0)	0 (8.5)	<0.001
Use of modified ultrafiltration	1190 (97.2)	944 (97.2)	255 (97.0)	0.97
Total UF volume (mL/kg)	93.8 (48.8)	92 (47)	102.9 (56.2)	<0.001
AUC < DO_2i^{400}	2065.2 (2847.1)	1979.6 (2630)	2652.6 (4087.3)	<0.001
AUC < DO_2i^{380}	1416.5 (2189.2)	1363.5 (2064)	1924.2 (3125.3)	<0.001
AUC < DO_2i^{360}	956.1 (1627.2)	927.3 (1453.5)	1219 (2286.5)	<0.001
AUC < DO_2i^{340}	644.1 (1033.2)	607.5 (954.1)	791.9 (1508.8)	<0.001
AUC < DO_2i^{320}	415.4 (714.5)	394.3 (666.9)	540.2 (954.6)	<0.001
AUC < DO_2i^{300}	285.5 (503.2)	275.5 (479.5)	315.3 (588.2)	0.001
AUC < DO_2i^{280}	223.4 (397.4)	214.9 (363.6)	255.7 (445)	0.006
AUC < DO_2i^{260}	167.6 (309.8)	162.6 (298.3)	192.8 (383.3)	0.02

Variables were compared using the Mann-Whitney U test or χ^2 test. Data are presented as median (IQR) because of non-normal distribution or as number (percent). AUC: area under the curve; CPB: cardiopulmonary bypass; DHCA: deep hypothermic circulatory arrest; DO_2i: indexed oxygen delivery; FFP: fresh frozen plasma; NP: nasopharyngeal; PO_2: partial pressure of oxygen; RBC: red blood cells; UF: ultrafiltration.
